# Associations between trunk-to-peripheral fat ratio and cardiometabolic risk factors in elderly Japanese men: baseline data from the Fujiwara-kyo Osteoporosis Risk in Men (FORMEN) study

**DOI:** 10.1186/s12199-021-00959-9

**Published:** 2021-03-20

**Authors:** Katsuyasu Kouda, Yuki Fujita, Kumiko Ohara, Takahiro Tachiki, Junko Tamaki, Akiko Yura, Jong-Seong Moon, Etsuko Kajita, Kazuhiro Uenishi, Masayuki Iki

**Affiliations:** 1 Department of Hygiene and Public Health, Kansai Medical University, 2-5-1 Shin-machi, Hirakata, Osaka, 573-1010 Japan; 2 Department of Public Health, Kindai University Faculty of Medicine, 377-2 Oono-higashi, Osaka-Sayama, Osaka, 589-8511 Japan; 3 Chukyo Gakuin University Faculty of Nursing, 2216 Tokicho, Mizunami, Gifu, 509-6192 Japan; 4 Department of Hygiene and Public Health, Osaka Medical College, 2-7 Daigakumachi, Takatsuki, Osaka, 569-8686 Japan; 5 Department of Nursing, Kio University, 4-2-2 Umami-naka, Koryo-cho, Nara, 635-0832 Japan; 6 Laboratory of Physiological Nutrition, Kagawa Nutrition University, 3-9-21 Chiyoda, Sakado, Saitama, 350-0288 Japan

**Keywords:** Body fat distribution, Densitometry, Epidemiology, Risk factors

## Abstract

**Background:**

Body mass-independent parameters might be more appropriate for assessing cardiometabolic abnormalities than weight-dependent indices in Asians who have relatively high visceral adiposity but low body fat. Dual-energy X-ray absorptiometry (DXA)-measured trunk-to-peripheral fat ratio is one such body mass-independent index. However, there are no reports on relationships between DXA-measured regional fat ratio and cardiometabolic risk factors targeting elderly Asian men.

**Methods:**

We analyzed cross-sectional data of 597 elderly men who participated in the baseline survey of the Fujiwara-kyo Osteoporosis Risk in Men (FORMEN) study, a community-based single-center prospective cohort study conducted in Japan. Whole-body fat and regional fat were measured with a DXA scanner. Trunk-to-appendicular fat ratio (TAR) was calculated as trunk fat divided by appendicular fat (sum of arm and leg fat), and trunk-to-leg fat ratio (TLR) as trunk fat divided by leg fat.

**Results:**

Both TAR and TLR in the group of men who used ≥ 1 medication for hypertension, dyslipidemia, or diabetes (“user group”; *N* = 347) were significantly larger than those who did not use such medication (“non-user group”; *N* = 250) (*P* < 0.05). After adjusting for potential confounding factors including whole-body fat, both TAR and TLR were significantly associated with low-density lipoprotein cholesterol, high-density lipoprotein cholesterol, triglyceride, fasting serum insulin, and the insulin resistance index in the non-user group and non-overweight men in the non-user group (*N* = 199).

**Conclusion:**

The trunk-to-peripheral fat ratio was associated with cardiometabolic risk factors independently of whole-body fat mass. Parameters of the fat ratio may be useful for assessing cardiometabolic risk factors, particularly in underweight to normal-weight populations.

## Background

Some individuals are not obese or overweight according to standard weight tables, but have metabolic abnormalities that are characteristically associated with obesity [1]. Such metabolically obese, normal-weight (MONW) individuals are very common in the general population [2, 3]. Most MONW individuals have increased abdominal adipose tissue, i.e., a metabolically adverse condition, with a relatively low body mass index (BMI) [4]. Data from the Obesity in Asia Collaboration showed that the absolute risk of diabetes or hypertension tended to be higher among Asians compared with Caucasians for any given level of BMI [5]. Associations between BMI and health risks differ in Asians compared with Europeans. For instance, Asians have a higher prevalence of type 2 diabetes but a lower BMI than Europeans [6]. One possible reason for this difference is that, for any given BMI, Asians have more visceral adiposity compared to Caucasians, and this difference may contribute to lipotoxicity and insulin resistance [7]. In fact, ethnic differences in visceral adiposity as measured by computed tomography have been reported [4].

Waist-to-hip ratio (WHR), a conventional weight-independent parameter and an index of abdominal fat distribution [8], was suggested to be a more appropriate index of cardiometabolic abnormalities than BMI for Asians [6]. Dual-energy X-ray absorptiometry (DXA)-measured trunk-to-peripheral fat ratio (a specific surrogate for visceral fat proportion) [9] is also a weight-independent index and a more precise method for measuring abdominal fat distribution than WHR [10]. Asians have been reported to have a higher DXA-measured trunk-to-peripheral fat ratio compared with non-Asians [11].

Associations between DXA-measured truncal fat proportion and cardiometabolic risk factors have also been reported for Europeans [12–14] and North Americans [15, 16]. However, only limited data are available on the relationship between DXA-measured truncal fat proportion and cardiometabolic risk factors in Asian adults, although a relationship between trunk-to-peripheral fat ratio and blood pressure in children has been reported [17]. Only one report from the Japanese Population-Based Osteoporosis (JPOS) Cohort Study has reported that DXA-measured trunk-to-peripheral fat ratio in women was associated with cardiometabolic variables [18]. However, no study has reported on associations between DXA-measured truncal fat proportion and cardiometabolic risk factors in elderly Asian men.

The present study aimed to investigate the relationship between DXA-measured trunk-to-peripheral fat ratio and cardiometabolic risk factors in community-dwelling elderly Japanese men using baseline data from the second Fujiwara-kyo Osteoporosis Risk in Men (FORMEN) study.

## Methods

### Study population

The FORMEN study is a large-scale community-based single-center prospective cohort study which aims to determine risk factors for osteoporotic fractures, prevent frailty, increase the number of healthy life years, and enhance the functioning and quality of life of elderly men in Nara Prefecture, Japan. Participants at the baseline survey were aged ≥ 65 years, lived at home, were able to walk without assistance from another person, and were able to provide self-reported information and written informed consent. The baseline survey of the first cohort of the FORMEN study was conducted from June 2007 to October 2008; details of the first cohort have been described elsewhere [19]. In the present study, the study population consisted of 599 elderly men who participated in the baseline survey of the second cohort, which was conducted from August 2019 to January 2020. After excluding 2 men with missing values for DXA-measured regional fat or blood test, the baseline data of 597 participants were subjected to cross-sectional analysis.

### Measurement of whole-body and regional fat

Whole-body and regional fat mass were measured with a single DXA scanner (QDR-4500A; Hologic Inc., Bedford, MA, USA) mounted on a mobile examination car in the same way as described previously [17, 18]. The same experienced medical radiology technician performed scans for all participants. Quality control checks of the DXA scanner were completed on a regular basis using several Hologic phantoms. Participants were asked to change into a gown for the scan, remove all metal objects (jewelry, zipper, belts, snaps, underwire bras, etc.) and their shoes, lay flat on the scanner table without a pillow in the dorsal position, and remain still and breath normally during the scan. After the scan, we obtained a whole-body posterior-anterior (PA) scan image of each participant. Arm, leg, and head regions in the whole-body PA scan image were isolated from the trunk region using the following standard manufacturer-recommended methods [10]: (a) the head region: the horizontal neck (shoulder) line just below the chin, (b) arm regions: the vertical shoulder line bisecting the shoulder joints, and (c) leg regions: the lower pelvic divider lines (two angled lines) bisecting both femoral necks. Ratios of trunk-to-peripheral fat were calculated using fat mass volume in the trunk, arm, and leg regions. Trunk-to-appendicular fat ratio (TAR) was calculated as trunk fat divided by appendicular fat (sum of arm and leg fat), and trunk-to-leg fat ratio (TLR) as trunk fat divided by leg fat.

### Measurements of body size and blood pressure

Body weight, height, and waist circumference were measured in light clothing with no shoes. BMI was calculated as weight divided by height squared (kg/m^2^). Overweight and underweight participants were identified using BMI cut-offs of 25 kg/m^2^ and 18.5 kg/m^2^, respectively. Systolic blood pressure and diastolic blood pressure were measured using an automatic oscillometric sphygmomanometer (BP-203i, OMRON COLIN, Tokyo, Japan) with an appropriate cuff size based on the upper arm circumference of each participant. Participants were relaxed and seated with legs uncrossed at an appropriate ambient temperature. Measurements were performed with the right arm supported at the level of the heart after resting for 5 min. The mean value of two readings was used for analysis.

### Lifestyle factors and medical history

Trained health care nurses interviewed participants based on answers to a self-administered questionnaire that covered lifestyle factors including smoking (current or non-smoker) and physical activity. Participants were asked to bring current prescriptions of medications to the baseline visit, and the nurses recorded the names and doses of the medications. Food frequency questionnaire (FFQ) for dietary intake of nutrients in Japan (FFQ for the Prevention and Management of Osteoporosis; FFQPOP [20]) was used to estimate dietary nutrient intake. Participants were asked to select a grade of frequency of food intake during the past 1 month. Responses to the questionnaire were also verified by dietitians during their interviews with participants. Intakes of nutrients and energy were estimated using the Standard Table of Food Composition in Japan [21]. Information on physical activity was obtained using the Japanese version of the International Physical Activity Questionnaire (IPAQ), which has been validated for Japanese adults aged 65 years and older [22]. Total weekly physical activity (metabolic equivalent of task (MET)-minutes/week) was estimated in accordance with official IPAQ guidelines [23].

### Biochemical analysis

Blood samples were obtained by vein puncture after an overnight fast for conventional biochemical tests. Levels of triglyceride (mg/dl) were determined by the enzymatic method (glycerol phosphate oxidase with glycerol blank) (Pureauto S TG-N, Sekisui Medical Co., Ltd., Tokyo, Japan); high-density lipoprotein (HDL; mg/dl) cholesterol by the Sekisui HDL direct method (Cholestest N HDL, Sekisui Medical Co., Ltd., Tokyo, Japan); low-density lipoprotein (LDL; mg/dl) cholesterol by the Sekisui LDL direct method (Cholestest LDL, Sekisui Medical Co., Ltd., Tokyo, Japan); total cholesterol (mg/dl) by the ultraviolet method with cholesterol dehydrogenase (T-CHO reagents, KL and “Kokusai”, Sysmex Corp., Kobe, Japan); fasting plasma glucose (mg/dl) by the ultraviolet method with hexokinase (CicaLiquid GLU, Kanto Chemical Co., Inc., Tokyo, Japan); Hemoglobin A1c (%, National Glycohemoglobin Standardization Program) by the latex aggregation immunoassay (RAPIDIA Auto HbA1c, Fujirebio Inc., Tokyo, Japan); fasting serum insulin (μU/ml) by the chemiluminescent enzyme immunoassay (Lumipulse Presto Insulin, Fujirebio Inc., Tokyo, Japan); aspartate aminotransferase (U/l) by the Japan Society of Clinical Chemistry recommended method (CicaLiquid AST, Kanto Chemical Co., Inc., Tokyo, Japan); alanine aminotransferase (U/l) by the Japan Society of Clinical Chemistry recommended method (CicaLiquid ALT, Kanto Chemical Co., Inc., Tokyo, Japan); and creatinine (mg/dl) by the enzymatic method (Determiner L CRE, Hitachi Chemical Diagnostics Systems Co., Ltd., Tokyo Japan). To estimate insulin resistance, homeostasis model assessment-insulin resistance (HOMA-IR) was calculated using fasting plasma glucose and fasting serum insulin [24]. Estimated glomerular filtration rate (eGFR; ml/min/1.73 m^2^) was calculated using the Modification of Diet in Renal Disease Study modified for Japanese individuals by the Japanese Society of Nephrology as follows: eGFR = 194 × serum creatinine^−1.094^ × age^−0.287^ [25].

### Statistical analyses

All statistical analyses were performed with SPSS Statistics Desktop for Japan, Version 22 (IBM Japan, Ltd., Tokyo, Japan). Frequency distributions for the biochemical tests were determined. We confirmed that levels of biochemical markers were distributed log-normally. Therefore, these values were logarithmically converted before statistical analyses, analyzed, and expressed as geometric means. Medication status might affect cardiometabolic variables. Thus, subjects were stratified by mediation status to evaluate the association between TAR/TLR and cardiometabolic variables. Comparisons between users of hypertension, dyslipidemia, or diabetes medication (“user group”) and those who did not use such medication (“non-user group”) were performed using the unpaired *t* test or Mann-Whitney *U* test. Correlations among cardiometabolic variables, potential confounders, and TAR/TLR were evaluated using Pearson’s correlation coefficients. Associations between TAR/TLR and cardiometabolic variables in the non-user or user group were evaluated using multiple regression analysis after adjusting for age, height, MET-minutes/week, current smoking, alcohol intake, energy intake, NaCl intake, and whole-body fat.

## Results

Table 1 shows the characteristics of men who used ≥ 1 medication for hypertension, dyslipidemia, or diabetes (user group) and those who did not use any medication (non-user group). Both TAR and TLR in the user group were significantly larger compared to those in the non-user group. Table 2 shows differences between the user and non-user groups according to the type of medication. Both TAR and TLR for each type of medication were significantly larger in the user group compared to the non-user group.

**Table 1:** Participant characteristics in the user group (use of one or more medication) and the non-user group

	Medication for hypertension, dyslipidemia, or diabetes		
	User, *N* = 347	Non-user, *N* = 250	*P*
Age (years)	74.0 ± 5.4	72.4 ± 5.2	< 0.01
Height (cm)	164.6 ± 6.0	165.2 ± 5.6	ns
Weight (kg)	64.4 ± 8.6	61.9 ± 8.3	< 0.01
Body mass index (kg/m^2^)	23.7 ± 2.7	22.7 ± 2.7	< 0.01
Overweight, *N* (%)	100 (29)	51 (20)	0.02
Underweight, *N* (%)	6 (2)	18 (7)	< 0.01
Waist circumference (cm)	86.9 ± 7.4	83.8 ± 7.9	< 0.01
Whole-body fat (kg)	13.9 ± 4.7	11.8 ± 4.4	< 0.01
Trunk fat (kg)	7.4 ± 3.0	6.1 ± 2.8	< 0.01
Arm fat (kg)	1.4 ± 0.5	1.3 ± 0.5	< 0.01
Leg fat (kg)	4.2 ± 1.5	3.8 ± 1.4	< 0.01
TAR	1.31 ± 0.37	1.19 ± 0.36	< 0.01
TLR	1.79 ± 0.57	1.61 ± 0.54	< 0.01
MET-minutes/week, median (25, 75%ile)	2106 (1025, 4176)	2523 (1067, 4718)	ns
Current smoker, *N* (%)	32 (9)	24 (10)	ns
Alcohol intake (kcal/day)	86 ± 131	97 ± 146	ns
Energy intake (kcal/day)	1693 ± 320	1716 ± 296	ns
NaCl intake (g/day)	10.0 ± 1.8	10.1 ± 1.9	ns
Systolic blood pressure (mmHg)	138.9 ± 16.9	139.5 ± 18.8	ns
Diastolic blood pressure (mmHg)	77.5 ± 9.9	80.5 ± 10.5	< 0.01
LDL cholesterol (mg/dl)	111.3 ^×^/_÷_ 1.3	119.4 ^×^/_÷_ 1.3	< 0.01
HDL cholesterol (mg/dl)	59.8 ^×^/_÷_ 1.3	63.6 ^×^/_÷_ 1.3	< 0.01
Total cholesterol (mg/dl)	196.8 ^×^/_÷_1.2	209.6 ^×^/_÷_ 1.2	< 0.01
Triglyceride (mg/dl)	100.7 ^×^/_÷_ 1.6	92.0 ^×^/_÷_ 1.6	0.02
Hemoglobin A1c (%)	6.0 ^×^/_÷_ 1.1	5.7 ^×^/_÷_ 1.1	< 0.01
Fasting serum insulin (mU/l)	4.8 ^×^/_÷_ 2.0	3.5 ^×^/_÷_ 1.8	< 0.01
Fasting plasma glucose (mg/dl)	106.1 ^×^/_÷_ 1.3	98.0 ^×^/_÷_ 1.2	< 0.01
HOMA-IR	1.3 ^×^/_÷_ 2.4	0.9 ^×^/_÷_ 2.0	< 0.01
Aspartate aminotransferase (U/l)	24.3 ^×^/_÷_ 1.3	24.1 ^×^/_÷_ 1.3	ns
Alanine aminotransferase (U/l)	19.7 ^×^/_÷_ 1.5	19.1 ^×^/_÷_ 1.4	ns
Estimated glomerular filtration rate (ml/min/1.73 m^2^)	64.9 ± 14.3	69.9 ± 13.2	< 0.01

Data are expressed as mean ± SD, median (25, 75%ile), geometric mean ×/÷ SD, or *N* (%)

Biochemical marker values were logarithmically converted and then statistically analyzed

*P* values were calculated using the unpaired *t* test or Mann-Whitney *U* test

*P* < 0.05 was considered statistically significant

MET-minutes/week was calculated using the International Physical Activity Questionnaire

*N*, number; ns, not significant; TAR, trunk-to-appendicular fat ratio; TLR, trunk-to-leg fat ratio; MET, metabolic equivalent of task; HDL, high-density lipoprotein; LDL, low-density lipoprotein; HOMA-IR, homeostasis model assessment-insulin resistance; SD, standard deviation

**Table 2:** Differences between user and non-user groups according to the type of medication

	Medication for								
	Hypertension			Dyslipidemia			Diabetes		
	User, *N* = 256	Non-user, *N* = 341	*P*	User, *N* = 167	Non-user, *N* = 430	*P*	User, *n* = 92	Non-user, *N* = 505	*P*
Age (years)	74.7 ± 5.5	72.3 ± 5.1	< 0.01	73.9 ± 5.6	73.1 ± 5.3	ns	73.6 ± 4.6	73.3 ± 5.5	ns
Height (cm)	164.0 ± 5.7	165.4 ± 5.9	< 0.01	165.0 ± 6.4	164.8 ± 5.6	ns	164.9 ± 5.7	164.8 ± 5.9	ns
Weight (kg)	64.5 ± 8.9	62.5 ± 8.2	< 0.01	64.7 ± 8.5	62.8 ± 8.5	0.02	64.2 ± 9.0	63.2 ± 8.5	ns
Body mass index (kg/m^2^)	23.9 ± 2.7	22.8 ± 2.7	< 0.01	23.7 ± 2.4	23.1 ± 2.8	0.01	23.6 ± 3.0	23.2 ± 2.7	ns
Overweight, *N* (%)	79 (31)	72 (21)	< 0.01	46 (28)	105 (24)	ns	24(26)	127 (25)	ns
Underweight, *N* (%)	3 (1)	21 (6)	< 0.01	3 (2)	21 (5)	ns	3 (3)	21 (4)	ns
Waist circumference (cm)	87.3 ± 7.4	84.3 ± 7.8	< 0.01	87.0 ± 7.0	85.1 ± 8.0	< 0.01	86.8 ± 7.9	85.4 ± 7.7	ns
Whole-body fat (kg)	14.3 ± 4.6	12.1 ± 4.6	< 0.01	14.0 ± 4.5	12.6 ± 4.7	< 0.01	13.0 ± 5.1	13.0 ± 4.6	ns
Trunk fat (kg)	7.7 ± 2.9	6.2 ± 2.9	< 0.01	7.5 ± 2.8	6.6 ± 3.0	< 0.01	7.0 ± 3.1	6.8 ± 2.9	ns
Arm fat (kg)	1.5 ± 0.6	1.3 ± 0.5	< 0.01	1.5 ± 0.5	1.3 ± 0.5	< 0.01	1.4 ± 0.6	1.4 ± 0.5	ns
Leg fat (kg)	4.3 ± 1.5	3.8 ± 1.4	< 0.01	4.2 ± 1.5	4.0 ± 1.5	0.03	3.9 ± 1.6	4.1 ± 1.5	ns
TAR	1.34 ± 0.37	1.21 ± 0.36	< 0.01	1.34 ± 0.36	1.23 ± 0.37	< 0.01	1.35 ± 0.42	1.25 ± 0.36	0.02
TLR	1.83 ± 0.58	1.63 ± 0.54	< 0.01	1.83 ± 0.56	1.67 ± 0.56	< 0.01	1.85 ± 0.65	1.69 ± 0.54	0.01
MET-minutes/week, median (25, 75%ile)	2118 (1188, 3801)	2502 (990, 4764)	ns	2070 (1097, 3792)	2337 (1017, 4632)	ns	1855 (1066, 3660)	2376 (1032, 4590)	ns
Current smoker, *N* (%)	23 (9)	33 (10)	ns	13 (8)	43 (10)	ns	12 (13)	44 (9)	ns
Alcohol intake (kcal/day)	92 ± 133	90 ± 141	ns	73 ± 111	97 ± 145	0.03	62 ± 93	96 ± 143	< 0.01
Energy intake (kcal/day)	1681 ± 330	1718 ± 295	ns	1679 ± 293	1712 ± 317	ns	1699 ± 350	1703 ± 303	ns
NaCl intake (g/day)	10.1 ± 1.8	10.0 ± 1.9	ns	10.0 ± 1.8	10.0 ± 1.9	ns	10.1 ± 1.8	10.0 ± 1.9	ns
Systolic blood pressure (mmHg)	140.0 ± 16.9	138.5 ± 18.2	ns	138.8 ± 17.4	139.3 ± 17.8	ns	134.8 ± 16.5	139.9 ± 17.8	0.01
Diastolic blood pressure (mmHg)	77.8 ± 10.1	79.5 ± 10.4	0.04	77.4 ± 10.0	79.3 ± 10.4	0.04	73.5 ± 9.8	79.7 ± 10.1	< 0.01
LDL cholesterol (mg/dl)	110.9 ^×^/_÷_ 1.3	117.5 ^×^/_÷_ 1.3	0.01	107.0 ^×^/_÷_ 1.3	117.7 ^×^/_÷_ 1.3	< 0.01	104.3 ^×^/_÷_ 1.3	116.6 ^×^/_÷_ 1.3	< 0.01
HDL cholesterol (mg/dl)	58.8 ^×^/_÷_ 1.3	63.4 ^×^/_÷_ 1.3	< 0.01	59.1 ^×^/_÷_ 1.2	62.3 ^×^/_÷_ 1.3	0.01	58.7 ^×^/_÷_ 1.3	61.9 ^×^/_÷_ 1.3	0.04
Total cholesterol (mg/dl)	195.6 ^×^/_÷_ 1.2	207.1 ^×^/_÷_ 1.2	< 0.01	191.9 ^×^/_÷_ 1.2	206.2 ^×^/_÷_ 1.2	< 0.01	188.1 ^×^/_÷_ 1.2	204.7 ^×^/_÷_ 1.2	< 0.01
Triglyceride (mg/dl)	101.5 ^×^/_÷_ 1.6	93.7 ^×^/_÷_ 1.6	0.04	105.2 ^×^/_÷_ 1.6	93.9 ^×^/_÷_ 1.6	< 0.01	110.7 ^×^/_÷_ 1.6	94.7 ^×^/_÷_ 1.6	< 0.01
Hemoglobin A1c (%)	5.9 ^×^/_÷_ 1.1	5.9 ^×^/_÷_ 1.1	ns	6.0 ^×^/_÷_ 1.1	5.8 ^×^/_÷_ 1.1	< 0.01	6.8 ^×^/_÷_ 1.1	5.7 ^×^/_÷_ 1.1	< 0.01
Fasting serum insulin (mU/l)	4.8 ^×^/_÷_ 2.0	3.8 ^×^/_÷_ 1.9	< 0.01	4.9 ^×^/_÷_ 1.9	4.0 ^×^/_÷_ 2.0	< 0.01	7.3 ^×^/_÷_ 2.6	3.8 ^×^/_÷_ 1.8	< 0.01
Fasting plasma glucose (mg/dl)	104.3 ^×^/_÷_ 1.3	101.4 ^×^/_÷_ 1.2	ns	105.2 ^×^/_÷_ 1.3	101.7 ^×^/_÷_ 1.2	ns	135.1 ^×^/_÷_ 1.4	97.6 ^×^/_÷_ 1.2	< 0.01
HOMA-IR	1.2 ^×^/_÷_ 2.4	1.0 ^×^/_÷_ 2.2	< 0.01	1.3 ^×^/_÷_ 2.2	1.0 ^×^/_÷_ 2.3	< 0.01	2.4 ^×^/_÷_ 3.2	0.9 ^×^/_÷_ 1.9	< 0.01
Aspartate aminotransferase (U/l)	24.1 ^×^/_÷_ 1.3	24.3 ^×^/_÷_ 1.3	ns	24.6 ^×^/_÷_ 1.3	24.0 ^×^/_÷_ 1.3	ns	23.0 ^×^/_÷_ 1.4	24.4 ^×^/_÷_ 1.3	0.04
Alanine aminotransferase (U/l)	19.4 ^×^/_÷_ 1.5	19.4 ^×^/_÷_ 1.4	ns	20.1 ^×^/_÷_ 1.5	19.2 ^×^/_÷_ 1.5	ns	20.0 ^×^/_÷_ 1.5	19.3 ^×^/_÷_ 1.4	ns
Estimated glomerular filtration rate (ml/min/1.73 m^2^)	64.0 ± 14.6	69.2 ± 13.3	< 0.01	62.7 ± 14.4	68.7 ± 13.6	< 0.01	64.7 ± 16.1	67.4 ± 13.7	ns

Data are expressed as mean ± SD, median (25, 75%ile), geometric mean ×/÷ SD, or *N* (%)

Biochemical marker values were logarithmically converted and then statistically analyzed

*P* values were calculated using the unpaired *t* test or Mann-Whitney *U* test. *P* < 0.05 was considered statistically significant

MET-minutes/week was calculated using the International Physical Activity Questionnaire

*N*, number; ns, not significant; TAR, trunk-to-appendicular fat ratio; TLR, trunk-to-leg fat ratio; MET, metabolic equivalent of task; HDL, high-density lipoprotein; LDL, low-density lipoprotein; HOMA-IR, homeostasis model assessment-insulin resistance; SD, standard deviation

Table 3 shows correlations between TAR/TLR and cardiometabolic variables, and between TAR/TLR and potential confounders. In the non-user group (*N* = 250), both TAR and TLR were significantly correlated with LDL cholesterol, HDL cholesterol, triglyceride, hemoglobin A1c, fasting serum insulin, fasting plasma glucose, and HOMA-IR. Similarly, both TAR and TLR in non-overweight men of the non-user group (*N* = 199) were significantly correlated with LDL cholesterol, HDL cholesterol, triglyceride, fasting serum insulin, and HOMA-IR. Both TAR and TLR also showed significant positive correlations with whole-body fat in the non-user group and non-overweight men of the non-user group.

**Table 3:** Correlations between TAR/TLR and cardiometabolic variables, and between TAR/TLR and potential confounders

	Age		Height		MET-minutes /week		Current smoker		Alcohol intake		Energy intake		NaCl intake		Whole-body fat		TAR		TLR	
	*r*	*P*	*r*	*P*	*r*	*P*	*r*	*P*	*r*	*P*	*r*	*P*	*r*	*P*	*r*	*P*	*r*	*P*	*r*	*P*
Non-user group, *N* = 250																				
Systolic blood pressure	0.06	ns	− 0.01	ns	0.15	0.01	0.03	ns	0.14	0.03	− 0.04	ns	− 0.02	ns	0.04	ns	0.04	ns	0.04	ns
Diastolic blood pressure	− 0.21	<0.01	0.14	0.03	0.07	ns	0.05	ns	0.11	ns	0.03	ns	− 0.02	ns	0.15	0.02	0.07	ns	0.06	ns
LDL cholesterol	− 0.16	0.01	0.11	ns	− 0.10	ns	0.07	ns	− 0.08	ns	− 0.01	ns	− 0.02	ns	0.15	0.02	0.22	<0.01	0.23	<0.01
HDL cholesterol	0.05	ns	− 0.02	ns	0.08	ns	− 0.17	<0.01	0.22	<0.01	− 0.10	ns	− 0.05	ns	− 0.41	<0.01	− 0.34	<0.01	− 0.31	<0.01
Total cholesterol	− 0.14	0.02	0.09	ns	− 0.03	ns	0.01	ns	0.02	ns	− 0.07	ns	− 0.05	ns	0.04	ns	0.13	0.04	0.15	0.02
Triglyceride	− 0.11	ns	0.07	ns	− 0.02	ns	0.11	ns	− 0.07	ns	0.04	ns	− 0.03	ns	0.44	<0.01	0.42	<0.01	0.42	<0.01
Hemoglobin A1c	− 0.03	ns	0.02	ns	0.03	ns	− 0.03	ns	− 0.14	0.03	0.03	ns	− 0.03	ns	0.10	ns	0.17	<0.01	0.19	<0.01
Fasting serum insulin	− 0.16	0.01	0.03	ns	− 0.14	0.03	0.01	ns	− 0.09	ns	− 0.02	ns	− 0.07	ns	0.49	<0.01	0.41	<0.01	0.39	<0.01
Fasting plasma glucose	− 0.07	ns	− 0.02	ns	− 0.07	ns	0.00	ns	0.01	ns	− 0.05	ns	0.00	ns	0.09	ns	0.19	<0.01	0.18	<0.01
HOMA-IR	− 0.16	0.01	0.02	ns	− 0.14	0.02	0.01	ns	− 0.08	ns	− 0.03	ns	− 0.06	ns	0.46	<0.01	0.41	<0.01	0.39	<0.01
Aspartate aminotransferase	0.09	ns	− 0.04	ns	0.02	ns	− 0.10	ns	0.14	0.03	− 0.13	0.04	− 0.05	ns	− 0.13	0.05	0.06	ns	0.07	ns
Alanine aminotransferase	− 0.11	ns	− 0.05	ns	0.02	ns	− 0.01	ns	− 0.07	ns	0.01	ns	− 0.14	0.03	0.11	ns	0.23	<0.01	0.22	<0.01
Estimated glomerular filtration rate	− 0.18	<0.01	− 0.05	ns	0.09	ns	0.05	ns	0.09	ns	0.06	ns	0.03	ns	− 0.06	ns	− 0.14	0.03	− 0.14	0.03
TAR	− 0.18	<0.01	0.00	ns	− 0.05	ns	0.03	ns	0.06	ns	− 0.06	ns	0.00	ns	0.47	<0.01	N/A		N/A	
TLR	− 0.19	<0.01	− 0.01	ns	− 0.03	ns	0.01	ns	0.06	ns	− 0.07	ns	0.01	ns	0.43	<0.01	N/A		N/A	
Non-overweight men of the non-user group, *N* = 199																				
Systolic blood pressure	0.12	ns	− 0.05	ns	0.11	ns	0.03	ns	0.19	0.01	− 0.09	ns	− 0.03	ns	0.11	ns	0.04	ns	0.03	ns
Diastolic blood pressure	− 0.20	<0.01	0.12	ns	0.06	ns	0.06	ns	0.15	0.04	0.00	ns	− 0.05	ns	0.21	<0.01	0.05	ns	0.04	ns
LDL cholesterol	− 0.13	ns	0.15	0.03	− 0.08	ns	0.07	ns	− 0.06	ns	0.01	ns	− 0.02	ns	0.27	<0.01	0.30	<0.01	0.30	<0.01
HDL cholesterol	0.07	ns	− 0.04	ns	0.08	ns	− 0.16	0.02	0.23	<0.01	− 0.07	ns	− 0.03	ns	− 0.38	<0.01	− 0.33	<0.01	− 0.30	<0.01
Total cholesterol	− 0.11	ns	0.12	ns	− 0.01	ns	0.01	ns	0.05	ns	− 0.07	ns	− 0.07	ns	0.18	0.01	0.21	<0.01	0.23	<0.01
Triglyceride	− 0.10	ns	0.08	ns	0.00	ns	0.11	ns	− 0.11	ns	− 0.04	ns	− 0.07	ns	0.43	<0.01	0.41	<0.01	0.41	<0.01
Hemoglobin A1c	− 0.03	ns	0.04	ns	0.03	ns	− 0.02	ns	− 0.14	0.05	0.01	ns	− 0.05	ns	0.06	ns	0.13	ns	0.13	ns
Fasting serum insulin	− 0.13	ns	0.05	ns	− 0.13	ns	0.01	ns	− 0.14	ns	− 0.05	ns	− 0.10	ns	0.44	<0.01	0.37	<0.01	0.33	<0.01
Fasting plasma glucose	− 0.05	ns	0.02	ns	− 0.10	ns	0.02	ns	− 0.05	ns	− 0.05	ns	− 0.01	ns	0.09	ns	0.14	0.05	0.12	ns
HOMA-IR	− 0.13	ns	0.05	ns	− 0.14	0.05	0.02	ns	− 0.13	ns	− 0.05	ns	− 0.09	ns	0.41	<0.01	0.36	<0.01	0.32	<0.01
Aspartate aminotransferase	0.11	ns	− 0.07	ns	0.02	ns	− 0.15	0.04	0.13	ns	− 0.12	ns	− 0.01	ns	− 0.13	ns	0.07	ns	0.08	ns
Alanine aminotransferase	− 0.07	ns	− 0.04	ns	− 0.02	ns	− 0.06	ns	− 0.07	ns	0.02	ns	− 0.12	ns	0.11	ns	0.23	<0.01	0.23	<0.01
Estimated glomerular filtration rate	− 0.13	ns	− 0.09	ns	0.07	ns	0.00	ns	0.09	ns	0.08	ns	0.00	ns	− 0.05	ns	− 0.17	0.02	− 0.18	0.01
TAR	− 0.15	ns	0.02	ns	− 0.06	ns	0.03	ns	0.04	ns	− 0.09	ns	0.00	ns	0.51	<0.01	N/A		N/A	
TLR	− 0.15	0.03	0.02	ns	− 0.05	ns	0.01	ns	0.04	ns	− 0.10	ns	0.01	ns	0.47	<0.01	N/A		N/A	

Biochemical marker values were logarithmically converted and then statistically analyzed

Pearson's correlation was used. *P* < 0.05 was considered statistically significant

MET-minutes/week was calculated using the International Physical Activity Questionnaire

MET, metabolic equivalent of task; *N*, number; ns, not significant; TAR, trunk-to-appendicular fat ratio; TLR, trunk-to-leg fat ratio; HDL, high-density lipoprotein; LDL, low-density lipoprotein; HOMA-IR, homeostasis model assessment-insulin resistance; N/A, not applicable

Table 4 shows associations between TAR/TLR and cardiometabolic variables after adjusting for potential confounders including whole-body fat. Even after adjusting for age, height, MET-minutes/week, current smoking, alcohol intake, energy intake, NaCl intake, and whole-body fat, both TAR and TLR were significantly associated with LDL/HDL cholesterol, triglyceride, fasting serum insulin, and HOMA-IR in the non-user group and non-overweight men of the non-user group.

**Table 4:** Associations between TAR/TLR and cardiometabolic variables after adjusting for potential confounders

	Total subjects, *N* = 597		Medications for hypertension, dyslipidemia, or diabetes					
			Users, *N* = 347		Non-users, *N* = 250		Non-overweight non-users, *N* = 199	
	Beta	*P*	Beta	*P*	Beta	*P*	Beta	*P*
TAR								
Systolic blood pressure	0.03	ns	0.04	ns	0.03	ns	− 0.02	ns
Diastolic blood pressure	0.00	ns	0.04	ns	− 0.02	ns	− 0.09	ns
LDL cholesterol	0.09	0.04	0.04	ns	0.19	< 0.01	0.22	< 0.01
HDL cholesterol	− 0.25	< 0.01	− 0.25	< 0.01	− 0.22	< 0.01	− 0.20	< 0.01
Total cholesterol	0.02	ns	− 0.02	ns	0.13	ns	0.16	ns
Triglyceride	0.32	< 0.01	0.33	< 0.01	0.28	< 0.01	0.27	< 0.01
Hemoglobin A1c	0.17	< 0.01	0.12	0.04	0.18	0.02	0.15	ns
Fasting serum insulin	0.25	< 0.01	0.23	< 0.01	0.23	< 0.01	0.19	< 0.01
Fasting plasma glucose	0.17	< 0.01	0.12	0.03	0.17	0.02	0.12	ns
HOMA− IR	0.25	< 0.01	0.23	< 0.01	0.25	< 0.01	0.20	< 0.01
TLR								
Systolic blood pressure	0.03	ns	0.04	ns	0.03	ns	− 0.02	ns
Diastolic blood pressure	− 0.01	ns	0.03	ns	− 0.03	ns	− 0.10	ns
LDL cholesterol	0.08	ns	0.02	ns	0.20	< 0.01	0.23	< 0.01
HDL cholesterol	− 0.24	< 0.01	− 0.25	< 0.01	− 0.20	< 0.01	− 0.18	0.01
Total cholesterol	0.02	ns	− 0.03	ns	0.16	0.03	0.18	0.03
Triglyceride	0.32	< 0.01	0.32	< 0.01	0.29	< 0.01	0.28	< 0.01
Hemoglobin A1c	0.17	< 0.01	0.12	0.03	0.19	< 0.01	0.15	ns
Fasting serum insulin	0.25	< 0.01	0.24	< 0.01	0.21	< 0.01	0.16	0.03
Fasting plasma glucose	0.17	< 0.01	0.12	0.03	0.17	0.02	0.09	ns
HOMA− IR	0.25	< 0.01	0.24	< 0.01	0.23	< 0.01	0.16	0.03

Biochemical marker values were logarithmically converted and then statistically analyzed

Multiple regression analysis was used after adjusting for age, height, MET-minutes/week, current smoker, alcohol intake, energy intake, NaCl intake, and whole-body fat

Beta represents the standard coefficient

*P* < 0.05 was considered statistically significant

MET-minutes/week was calculated using the International Physical Activity Questionnaire

*N*, number; ns, not significant; TAR, trunk-to-appendicular fat ratio; TLR, trunk-to-leg fat ratio; HDL, high-density lipoprotein; LDL, low-density lipoprotein; HOMA-IR, homeostasis model assessment-insulin resistance; MET, metabolic equivalent of task

Table 5 shows associations between TAR/TLR and cardiometabolic variables after adjusting for potential confounders including whole-body fat according to the type of medication. There were significant inverse associations between TAR/TLR and HDL cholesterol for each type of medication in the non-user group. In addition, there were significant positive associations between TAR/TLR and LDL cholesterol, triglyceride, hemoglobin A1c, fasting serum insulin, fasting plasma glucose, and HOMA-IR for each type of medication in the non-user group.

**Table 5:** Associations between TAR/TLR and cardiometabolic variables after adjusting for potential confounders according to the type of medication

	Medication for											
	Hypertension				Dyslipidemia				Diabetes			
	User, *N* = 256		Non-user, *N* = 341		User, *N* = 167		Non-user, *N* = 430		User, *N* = 92		Non-user, *N* = 505	
	Beta	*P*	Beta	*P*	Beta	*P*	Beta	*P*	Beta	*P*	Beta	*P*
TAR												
Systolic blood pressure	− 0.01	ns	0.05	ns	0.17	0.04	− 0.02	ns	0.11	ns	0.04	ns
Diastolic blood pressure	− 0.01	ns	0.00	ns	0.13	ns	− 0.04	ns	0.00	ns	0.04	ns
LDL cholesterol	0.01	ns	0.18	< 0.01	0.04	ns	0.13	0.01	0.10	ns	0.12	0.02
HDL cholesterol	− 0.24	< 0.01	− 0.25	< 0.01	− 0.17	0.03	− 0.26	< 0.01	− 0.31	< 0.01	− 0.23	< 0.01
Total cholesterol	− 0.04	ns	0.11	ns	0.05	ns	0.03	ns	0.04	ns	0.04	ns
Triglyceride	0.28	< 0.01	0.34	< 0.01	0.34	< 0.01	0.29	< 0.01	0.39	< 0.01	0.29	< 0.01
Hemoglobin A1c	0.17	< 0.01	0.17	< 0.01	0.12	ns	0.17	< 0.01	0.03	ns	0.16	< 0.01
Fasting serum insulin	0.24	< 0.01	0.24	< 0.01	0.26	< 0.01	0.24	< 0.01	0.21	ns	0.21	< 0.01
Fasting plasma glucose	0.16	0.02	0.16	0.01	0.17	0.04	0.15	< 0.01	0.04	ns	0.14	< 0.01
HOMA-IR	0.24	< 0.01	0.25	< 0.01	0.27	< 0.01	0.24	< 0.01	0.18	ns	0.22	< 0.01
TLR												
Systolic blood pressure	− 0.01	ns	0.06	ns	0.18	0.02	− 0.02	ns	0.11	ns	0.04	ns
Diastolic blood pressure	− 0.02	ns	0.00	ns	0.13	ns	− 0.05	ns	− 0.01	ns	0.03	ns
LDL cholesterol	0.00	ns	0.19	< 0.01	0.02	ns	0.13	0.01	0.10	ns	0.11	0.03
HDL cholesterol	− 0.23	< 0.01	− 0.24	< 0.01	− 0.17	0.03	− 0.25	< 0.01	− 0.32	< 0.01	− 0.22	< 0.01
Total cholesterol	− 0.05	ns	0.13	0.04	0.02	ns	0.05	ns	0.03	ns	0.05	ns
Triglyceride	0.27	< 0.01	0.35	< 0.01	0.33	< 0.01	0.30	< 0.01	0.40	< 0.01	0.29	< 0.01
Hemoglobin A1c	0.17	< 0.01	0.17	< 0.01	0.14	ns	0.17	< 0.01	0.04	ns	0.16	< 0.01
Fasting serum insulin	0.26	< 0.01	0.22	< 0.01	0.28	< 0.01	0.23	< 0.01	0.21	0.05	0.21	< 0.01
Fasting plasma glucose	0.17	< 0.01	0.14	0.02	0.18	0.03	0.15	< 0.01	0.04	ns	0.14	< 0.01
HOMA-IR	0.26	< 0.01	0.23	< 0.01	0.28	< 0.01	0.24	< 0.01	0.19	ns	0.22	< 0.01

Biochemical marker values were logarithmically converted and then statistically analyzed

Multiple regression analysis was used after adjusting for age, height, MET-minutes/week, current smoker, alcohol intake,

energy intake, NaCl intake, and whole-body fat

Beta represents the standard coefficient

*P* < 0.05 was considered statistically significant

MET-minutes/week was calculated using the International Physical Activity Questionnaire

*N*, number; ns, not significant; TAR, trunk-to-appendicular fat ratio; TLR, trunk-to-leg fat ratio; HDL, high-density lipoprotein; LDL, low-density lipoprotein; HOMA-IR, homeostasis model assessment-insulin resistance; MET, metabolic equivalent of task

## Discussion

The present cross-sectional study showed significant associations between trunk-to-peripheral fat ratio and cardiometabolic variables (LDL cholesterol, HDL cholesterol, triglyceride, hemoglobin A1c, fasting serum insulin, fasting plasma glucose, and HOMA-IR) in elderly Japanese men who did not use hypertension, dyslipidemia, or diabetes medication. These associations remained significant even after whole-body fat was accounted for, indicating that trunk-to-peripheral fat ratio, independently of overall fat mass, was associated with cardiometabolic variables. In addition, these associations were also observed among non-overweight participants. These results indicate that a large volume of body fat may not be a prerequisite for the development of cardiometabolic abnormalities, and weight-independent indices (non-weight-reliant indices) such as regional fat ratios may provide additional information for the evaluation of health risks.

A previous cross-sectional study from a representative Japanese female population (the JPOS study) reported that TAR was significantly correlated with LDL cholesterol, HDL cholesterol, and hemoglobin A1c levels and that the relationships between fat ratio and cardiometabolic variables were independent of relationships between fat volume and cardiometabolic variables [18]. Our present results from elderly Japanese men are consistent with results from that study. Thus, DXA-measured fat mass-independent indices (e.g., TAR and TLR) may be useful for assessing cardiometabolic disease risk, particularly in underweight to normal-weight populations. Trunk-to-peripheral fat ratio may also allow the characterization of MONW individuals, who are common in Asian population.

Although BMI is the most common parameter related to adiposity for all age groups, it reflects total fat mass rather than body fat distribution. On the other hand, WHR is a weight-independent index which more accurately describes abdominal fat distribution than BMI [8, 26, 27]. WHR is reportedly an alternative anthropometric index of obesity that overcomes the limitations of BMI, and predicts the clustering of cardiovascular risk factors in addition to weight-based indices such as BMI [28–30]. Previous studies in normal-weight populations found that high WHR levels were associated with insulin resistance markers and cardiovascular disease risk factors [31, 32]. Therefore, WHR might be a more appropriate index for evaluating cardiometabolic disease risk in Asian people who have lower BMI levels than those of European descent [6]. However, WHR cannot distinguish between fat mass and lean mass and can only serve as a surrogate for the distribution of detrimental visceral fat, beneficial gluteal fat, and muscle [27]. On the other hand, DXA devices are capable of measuring body composition (fat mass, lean mass, and bone mass) of the entire body or specific body regions (arm, leg, and trunk) with high precision, low scanning time, and low radiation exposure [33]. DXA-measured regional fat mass can be used to assess fat distribution using the trunk-to-peripheral fat ratio. DXA-measured trunk fat consists of visceral and subcutaneous fat, while arm or leg fat does not include visceral fat. Therefore, TAR and TLR reveal the relative amount of visceral adipose tissue as weight-independent indices. Body composition-based DXA-measured fat ratio is a more precise method for measuring the distribution of body fat than conventional anthropometric indices such as WHR.

Although the pathophysiological background underlying the associations between trunk-to-peripheral fat ratio and cardiometabolic variables was not explored in the present study, the associations might be attributed to differences between visceral and subcutaneous adipose tissue. Subcutaneous adipose tissue acts as a “protective metabolic sink,” metabolizing and storing excess free fatty acids and storing the bulk of body energy [34]. When the extra energy is channeled into subcutaneous adipose tissue, individuals with a positive energy balance would be protected against the development of metabolic syndrome. However, in individuals who lack subcutaneous adipose tissue and thus have a limited ability to store excess energy, surplus triglycerides are deposited at undesirable sites (e.g., visceral adipose tissue), a phenomenon referred to as ectopic fat deposition [34]. Given the unfavorable effects of ectopic fat deposits, the presence of subcutaneous adipose tissue may contribute to a reduced risk of various cardiometabolic outcomes by curbing ectopic fat deposition. Thus, the ratio of trunk fat (including visceral and subcutaneous fat) to peripheral fat (including subcutaneous fat) may be useful for evaluating cardiometabolic disease risk.

This study has several strengths. First, DXA can be used to determine the bone and soft tissue composition of the whole body and subregions such as the arms, legs, and the trunk and is a simple and safe technique that can be used for children and the old and frail [10]. Furthermore, the precision of all DXA measurements has been reported to be excellent [10]. Therefore, DXA is increasingly being used as a criterion or reference for comparison with other body composition measurements, such as the impedance method [35]. However, DXA-based body fat measurement is expensive and not suitable for assessing cardiovascular risk of very common lifestyle-related diseases. Second, this is the first report on relationships between trunk-to-peripheral fat ratio and cardiometabolic risk factors among Japanese elderly men who tend to have more visceral adiposity than European people. Third, the FORMEN study had a relatively large sample size. Fourth, the study was conducted as part of an ongoing cohort study, allowing for further follow-up studies with the same study participants. Finally, this was a single-center study, and thus, there was no inter-center variation.

There are also some limitations worth noting. First, participants were not a random sample, but rather volunteers living in a certain region of Japan, and thus may not be representative of the entire Japanese population. Older adults who volunteer and engage in more volunteer activities have been reported to be more likely to have higher levels of well-being, including self-rated health, functional dependency, and depressive symptomatology [36]. Individuals with serious diseases may also have been less likely to participate in the present study. Second, the cross-sectional design did not allow us to determine causal relationships between predictors and outcomes.

## Conclusion

The trunk-to-peripheral fat ratio, a body mass-independent index, was associated with cardiometabolic variables in elderly Japanese men. Moreover, these associations were also observed among non-overweight men and were independent of associations between whole-body fat and cardiometabolic variables. Fat ratio parameters may be useful for assessing cardiometabolic risk factors, particularly in underweight to normal-weight populations. The trunk-to-peripheral fat ratio may also allow for the characterization of MONW individuals who are very common in Asian populations.

## Data Availability

The datasets generated during the current study are not publicly available, but are available from the corresponding author on reasonable request.

## Abbreviations

BMI: Body mass index
DXA: Dual-energy X-ray absorptiometry
FFQ: Food frequency questionnaire
FFQPOP: FFQ for the Prevention and Management of Osteoporosis
FORMEN: Fujiwara-kyo Osteoporosis Risk in Men
HDL: High-density lipoprotein
HOMA-IR: Homeostasis model assessment-insulin resistance
IPAQ: International Physical Activity Questionnaire
JPOS: Japanese Population-Based Osteoporosis
LDL: Low-density lipoprotein
MET: Metabolic equivalent of task
MONW: Metabolically obese, normal-weight
TAR: Trunk-to-appendicular fat ratio
TLR: Trunk-to-leg fat ratio
WHR: Waist-to-hip ratio
